# Higher High-Sensitivity C Reactive Protein is Associated with Future Premature Ventricular Contraction: a Community Based Prospective Cohort Study

**DOI:** 10.1038/s41598-018-22868-8

**Published:** 2018-03-26

**Authors:** Yue Chen, Shouling Wu, Wenyu Li, Binhao Wang, Xu Han, Yiheng Yang, Xumin Guan, Haixu Yu, Bin Waleed Khalid, Huihua Li, Yunlong Xia

**Affiliations:** 1 Department of cardiology, First Affiliated Hospital of Dalian Medical University, Dalian, 116011 China; 2 Department of cardiology, Kailuan Hospital, Hebei United University, Tangshan, China

## Abstract

We aimed to determine whether hs-CRP is a predictor of future premature ventricular contraction (PVC) events in a community based population. A total of 101,510 participants were recruited at baseline (2006–2007). The follow-up visits were conducted every two years. Participants who were free from PVC at baseline and achieved the fourth visit, or diagnosed of PVC during the subsequent visits were included for analyses. Diagnosis of PVC was based on standard supine resting, 10-s 12-lead ECG. Cox regression was applied to evaluate the association between quartiles of hs-CRP and the incidence of PVCs. 60710 participants (male: 79.9%, mean age 49.4 years) were included for analyses. During a mean follow-up of 74.9 ± 7.4 months, 908 (1.5%) participants were diagnosed with PVC. Participants of the highest quartile of hs-CRP had significantly increased risk of PVC events as compared with the lowest quartile (HR 1.36; 95% CI 1.12–1.66); and stratified analyses showed similar result in males (HR 1.45; 95% CI 1.16–1.80), but not in females (HR 1.12; 95% CI 0.71–1.79). Moreover, elevated serum hs-CRP was associated with future PVC in participants without history of myocardial infarction or stroke (HR 1.34; 95% CI 1.09–1.65). Elevated hs-CRP was an independent predictor of PVC in Chinese population, especially in men.

## Introduction

Premature ventricular contractions (PVC) have been reported as a frequent arrhythmia, with the prevalence of up to 50% in asymptomatic people depending on the different populations evaluated^1,2^. The incidence of PVC varies from 1% to over 10% in healthy population, and tents to be higher in older people^3,4^. Accumulating evidence suggests that patients with PVC are at higher risk for many adverse cardiovascular events, such as impaired ventricular systolic function or congestive heart failure (CHF), overall cardiac mortality, as well as sudden death^5,6^. Indeed, PVC has been considered as an equivalent risk factor for many other risk factors for CHF^7^. Particularly, PVCs detected on 12-lead electrocardiogram (ECG) was demonstrated to have a relatively high sensitivity to predict frequent PVCs detected from Holter monitoring^8,9^. Despite the clinical findings that PVC increases the risk of adverse cardiovascular outcomes, the potential mechanisms underlying this association remain to be determined. Many mechanisms, including myocardial fibrosis, electrophysiological and structural remodeling and inflammation^10^ have been involved. However, the association between systematic inflammation and incidence of PVC has not been well evaluated.

As a classic marker of systematic inflammation, high-sensitive c reactive protein (hs-CRP) has been linked to many chronic disorders, such as hypertension, metabolic syndrome, coronary artery disease (CAD), myocardial infarction, and stroke^11–13^. In view of the fact that hs-CRP has been involved in the pathogenesis of many cardiovascular events, we hypothesized that elevated hs-CRP may predict PVC events. Therefore, in this community based prospective cohort study, we evaluated the effects of baseline hs-CRP level on the incidence of future PVC events. Stratified analyses were also performed to evaluate the potential influence of gender and other clinical characteristics on the association between hs-CRP and PVC risk.

## Methods

### Study design and population

**Figure 1:**
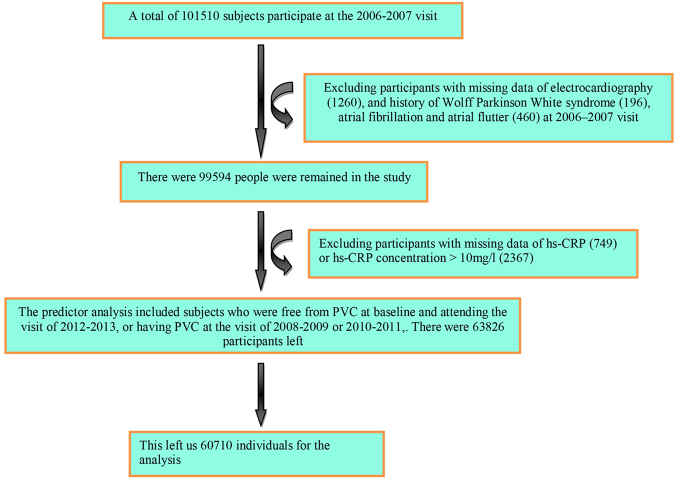
Flow chart of the participants’ enrollment process. hs-CRP, high-sensitivity c reactive protein; PVC, premature ventricular contraction.

This study was a community-based prospective study derived from the Kailuan Cohort Study. The Kailuan Cohort consisted of 101,510 subjects (81,110 males and 20,400 females) who were mainly the employee and retirees of Kailuan (Group) Co. Ltd, a large coal mine industry in Hebei Province, China. The baseline data were obtained from an health checkup of the participants. The follow-up visits were conducted every two years. All participants underwent questionnaire assessment, physical examination, and laboratory assessment. Standard protocols were used in all of the measurements as described earlier^14,15^. We enrolled subjects who were free from PVC at baseline and achieved the fourth visit, or diagnosed of PVC during the subsequent visits were included for analyses. Exclusion criteria included: (1) missing data of ECG or hs-CRP, (2) history with atrial fibrillation/flutter or Wolff Parkinson White (WPW) syndrome at baseline, (3) PVC was diagnosed in the first health examination, (4) participants with hs-CRP concentration >10 mg/l, which may be reflective of an acute inflammation^16^ (Fig. 1). The baseline characteristics of participants were presented based on the first examination of 2006 to 2007 in our research. The study was conducted in compliance with the law protecting personal data in accordance with the guidelines of Helsinki declaration. The study was approved by the Ethics Committee of Kailuan General Hospital, Written informed consent was obtained from all the participants.

### Assessment of Variables

Information on demographic and socioeconomic variables was collected via a questionnaire, including age, sex, marital status, medical history, family medical history, education level, average income of each family member, alcohol consumption, smoking status, physical activity, sleeping time quality. Smoking status was classified as never, former or current according to self-reported information. In the present study, we defined smokers as those who were current smokers. Hypertension was defined as presence of a history of hypertension, using antihypertensive medications, a systolic blood pressure > = 140 mmHg, or a diastolic pressure > = 90 mmHg. Diabetes mellitus (DM) was defined as a self-reported disease or the use of insulin, or oral hypoglycemic agents or FBG ≥ 7 mmol/L. Body weights (accurate to 0.1 kg) and heights (accurate to 0.1 cm) were measured, and the body mass indexes (BMI) were calculated. The estimated glomerular filtration rate (eGFR), which was calculated by the Modification of Diet in Renal Disease (MDRD) formula as follows: 186 × (serum creatinine^−1.154^) × (age^−0.203^) × (0.742 if female), with the serum creatinine concentration expressed in milligram per deciliter (mg/dl)^17^.

### Anthropometric measurements

Height and weight were measured while subjects wearing light clothing without shoes and hats. Height was measured to the nearest 0.1 cm using a portable stadiometer and weight was measured to the nearest 0.1 kg using calibrated platform scales. Blood pressure (BP) was measured to the nearest 1 mm Hg using mercury sphygmomanometers following the standard recommended procedures. Two readings each of systolic and diastolic blood pressures were recorded at 5-min intervals. The average of two readings was used for subsequent analysis. If the 2 measurements differed by more than 5 mmHg, an additional reading was taken. 12-lead electrocardiography was measured at rest for 5 minutes for the detection of arrhythmias including PVC.

### Laboratory tests

Blood samples were drawn from the antecubital vein in the morning at the fasting state and stored in vacuum tubes containing EDTA (Ethylene Dia mine Tetraacetic Acid), and all the blood tests were done at the central laboratory of the Kailuan hospital. Serum hs-CRP, creatinine, uric acid, total cholesterol (TC), triglycerides (TG), high-density lipoprotein cholesterol (HDL-C), low-density lipoprotein cholesterol (LDL-C), and fasting blood glucose (FBG) were assessed. Hs-CRP was measured by high-sensitivity nephelometry assay (Cias Latex CRP-H, Kanto Chemical Co. Inc, Tokyo, Japan).

### Statistical Analysis

Statistical analyses were performed using SAS software, version 9.1 (SAS Institute, Cary, North Carolina, USA) and SPSS 13.0 (SPSS Inc, Chicago, IL). Continuous variables were described by mean ± standard deviation (SD) and compared using one-way ANOVA analysis among multiple groups. Categorical variables were described by percentages and compared via Chi-Squared tests. Hs-CRP was divided into 4 categories according to the quartile^18^. Hazards ratio (HR) and 95% confidence intervals (CI) of incident PVC were estimated according to different hs-CRP concentrations and 1-SD increment of hs-CRP with Cox regression models. A two-tailed p value of <0.05 was considered statistically significant.

## Results

A total of 60710 participants (46700 men, 79.9%) with a mean age of 49.36 ± 11.56 years old were finally included in the analyses. The subjects were categorized into four groups according to the quartiles of hs-CRP concentrations. The demographic and clinical characteristics of the subjects are summarized in Table 1. In the present study, thresholds dividing the first and second, second and third, and third and fourth hs-CRP quartiles were 0.29, 0.71 and 1.80 mg/l for the whole population, 0.28, 0.77 and 2.1 mg/l for women, and 0.29, 0.70 and 1.73 mg/l for men. SDs of hs-CRP concentration for was 2.02 mg/l, 1.99 mg/l and 2.12 mg/l for the overall participants, women and men, respectively. Male subjects were in higher proportion (76.9%) in the study. Compared with the first hs-CRP quartile, Participants of the higher quartile of hs-CRP were associated with higher prevalence of cardiovascular risk factors, including hypertension, DM, MI, stroke, high BMI, advanced age, and elevated concentrations of systolic BP, diastolic BP, FBG.

**Table 1:** Baseline characteristics of participants by quartiles of hs-CRP

characteristics	Total	Quartiles of hs-CRP				P value
		<0.29	0.29 to 0.71	0.71 to 1.8	>1.80	
Age, ys	49.36 ± 11.56	47.04 ± 10.92	48.60 ± 11.41	49.66 ± 11.63	52.16 ± 11.68	<0.001
Male, n%	46700(76.9)	11708(76.6)	11866(78.5)	11941(78.6)	11185(74.0)	<0.001
BMI	25.10 ± 3.45	23.95 ± 3.22	24.95 ± 3.26	25.66 ± 3.34	25.85 ± 3.63	<0.001
Smoking, n%	20561(34.5)	5072(33.3)	5546(36.7)	5448(35.9)	4495(31.8)	<0.001
history of DM	4962(8.2)	848(5.5)	1026(6.8)	1393(9.2)	1695(11.2)	<0.001
history of HTN	25130(41.4)	5251(34.4)	5805(23.1)	6733(44.3)	7341(48.5)	<0.001
history of MI	626(1.0)	94(0.6)	158(1.0)	186(1.2)	188(1.3)	<0.001
history of stroke	1021(1.7)	132(0.9)	242(1.6)	294(1.9)	353(2.5)	<0.001
Systolic BP, mmHg	129.39 ± 20.08	126.18 ± 18.96	128.19 ± 19.47	130.91 ± 20.24	132.32 ± 21.01	<0.001
Diastolic BP, mmHg	83.18 ± 11.53	81.90 ± 11.28	82.59 ± 11.30	83.98 ± 11.55	84.25 ± 11.84	<0.001
FBG, mmol/L	5.40 ± 1.52	5.25 ± 1.25	5.35 ± 1.42	5.47 ± 1.58	5.52 ± 1.78	<0.001
TC, mmol/L	4.94 ± 1.16	4.78 ± 1.17	4.98 ± 1.14	5.02 ± 1.15	4.99 ± 1.14	<0.001
TG, mmol/L	1.69 ± 1.40	1.54 ± 1.32	1.62 ± 1.32	1.80 ± 1.48	1.81 ± 1.45	<0.001
LDL cholesterol, mmol/L	2.37 ± 0.89	2.33 ± 0.75	2.43 ± 0.79	2.47 ± 0.80	2.23 ± 1.16	<0.001
HDL cholesterol, mmol/L	1.54 ± 0.39	1.58 ± 0.40	1.53 ± 0.38	1.52 ± 0.39	1.54 ± 0.40	<0.001
Uric acid, mg/dL	4.80 ± 1.38	4.52 ± 1.23	4.82 ± 1.35	4.99 ± 1.42	4.86 ± 1.47	<0.001
Creatinine, umol/L	91.49 ± 29.99	91.81 ± 30.75	92.27 ± 27.16	92.68 ± 30.76	89.19 ± 30.98	<0.001
eGFR, ml/min*1.73 m2	84.54 ± 25.51	85.65 ± 25.15	84.21 ± 24.18	83.67 ± 24.78	84.62 ± 27.75	<0.001
**Medications, n(%)**						
ACEI	374(0.6)	46(0.3)	73(0.5)	124(0.8)	131(0.9)	<0.001
ARB	105(0.2)	17(0.1)	17(0.1)	27(0.2)	44(0.3)	<0.001
CCB	643(1.1)	61(0.4)	128(0.9)	212(1.4)	242(1.6)	<0.001
β-R inhibitor	203(0.3)	11(0.1)	38(0.3)	70(0.5)	84(0.6)	<0.001
PVC, n%	908(1.5)	179(1.2)	206(1.4)	224(1.5)	299(2.0)	<0.001

BMI, body mass index; HIN, hypertension; MI, myocardial infarction; DM, diabetes melitus; BP, blood pressure; FBG, fasting blood glucose; TC, Total cholesterol; TG, Triglyceride; LDL, low-density lipoprotein; HDL, high-density lipoprotein; eGFR, estimated glomerular filtration rate; ACEI, angiotensin-converting enzyme inhibitor; ARB, angiotensin receptor blocker; CCB, calcium channel blockers; PVC, premature ventricular contraction; Hs-CRP, high-sensitivity c reactive protein.

**Figure 2:**
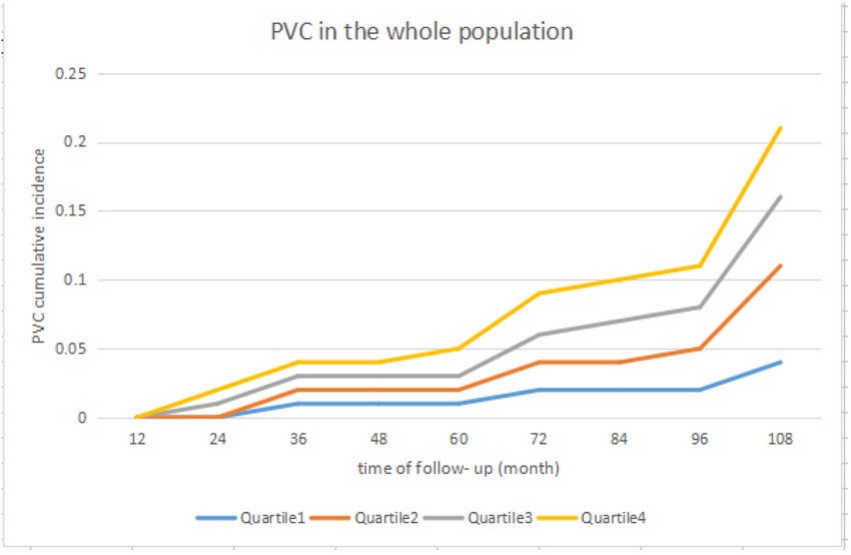
The cumulative incidence of PVC according to the quartiles of the baseline hs-CRP. PVC, premature ventricular contraction.

During a mean follow-up of 74.9 ± 7.4 months, 908(1.5%) participants were diagnosed of PVC. The incidence of PVC increased with increment of baseline hs-CRP concentration in total subjects and in men. From the first to the fourth hs-CRP quartile, the incidence of PVC was 1.2%, 1.4%, 1.5% and 2.0% in all participants, 1.2%, 1.3%, 1.6% and 2.2% in men, and 1.1%, 1.3%, 1.2% and 1.2% in women, respectively. The significant independent association between baseline hs-CRP and PVC occurrence was indicated by multivariable Cox regression analysis after adjusted for various clinical covariates. Participants of the highest quartile of hs-CRP had significantly increased risk of PVC events as compared with the lowest quartile (HR 1.36; 95% CI 1.12–1.66); and stratified analyses showed similar result in men (HR 1.36; 95% CI 1.12–1.66), but not in women (HR 1.12; 95% CI 0.71–1.79). HRs for associations between hs-CRP with PVC occurrence were also standardized to SD of hs-CRP concentration, which were 1.12 (1.05–1.19), 1.14 (1.06–1.22) and 1.02 (0.87–1.20), respectively. Additionally, we conducted sensitivity analyzes to evaluate the robustness of our findings. A similar result was presented in the participants without history of myocardial infarction or stroke (HR 1.34; 95% CI 1.09–1.65). (Table 2, Fig. 2).

**Table 2:** Associations between baseline hs-CRP and PVC risk: results of Cox-regression test.

	Quartiles of hs-CRP				P trend	Increase per SD	P value
	<0.29	0.29 to 0.71	0.71 to 1.80	>1.80			
**Total**							
Subjects	15017	14908	14964	14823			
PVC	179(1.2)	206(1.4)	224(1.5)	299(2.0)			
Model 1	1(ref)	1.20(0.98–1.46)	1.29(1.06–1.57)	1.78(1.47–2.14)	<0.001	1.23(1.17–1.30)	<0.001
Model 2	1(ref)	1.11(0.91–1.36)	1.15(0.95–1.40)	1.46(1.21–1.77)	<0.001	1.15(1.09–1.22)	<0.001
Model 3	1(ref)	1.108(0.90–1.36)	1.14(0.94–1.40)	1.36(1.12–1.66)	0.001	1.12(1.05–1.19)	0.001
Sensitivity analysis							
Model 4	1(ref)	1.13(0.92–1.39)	1.18(0.96–1.44)	1.36(1.11–1.67)	0.001	1.11(1.04–1.18)	0.001
Model 5	1(ref)	1.15(0.93–1.41)	1.19(0.97–1.46)	1.34(1.09–1.65)	0.002	1.11(1.04–1.18)	0.002
	**Quartiles of hs-CRP**				**P trend**	**Increase per SD**	**P value**
	**= < 0.29**	**0.29–0.7**	**0.70–1.73**	**>1.73**			
**Men**							
Subjects	11708	11745	11587	11660			
PVC	142(1.2)	156(1.3)	180(1.6)	253(2.2)			
Model 1	11708	1.14(0.91–1.44)	1.34(1.08–1.67)	1.92(1.56–2.36)	<0.001	1.26(1.19–1.34)	<0.001
Model 2	1(ref)	1.06(0.84–1.33)	1.21(0.97–1.50)	1.55(1.26–1.91)	<0.001	1.17(1.10–1.24)	<0.001
Model 3	1(ref)	1.06(0.84–1.34)	1.19(0.95–1.49)	1.45(1.16–1.80)	<0.001	1.14(1.06–1.22)	<0.001
**Sensitivity analysis**							
Model 4	1(ref)	1.09(0.86–1.38)	1.23(0.98–1.55)	1.46(1.16–1.82)	0.001	1.14(1.06–1.22)	<0.001
Model 5	1(ref)	1.11(0.88–1.41)	1.27(1.01–1.60)	1.45(1.15–1.82)	<0.001	1.14(1.06–1.22)	<0.001
	**Quartiles of hs-CRP**				**P trend**	**Increase per SD**	**P value**
	** = < 0.28**	**0.28 to 0.77**	**0.77 to 2.10**	**>2.10**			
**Women**							
Subjects	3510	3499	3543	3458			
PVC	37(1.1)	47(1.3)	41(1.2)	52(1.2)			
Model 1	1(ref)	1.26(0.82–1.93)	1.06(0.68–1.66)	1.42(0.93–2.17)	0.097	1.12(0.98–1.28)	0.097
Model 2	1(ref)	1.19(0.78–1.84)	0.97(0.62–1.52)	1.26(0.82–1.94)	0.270	1.08(0.94–1.24)	0.270
Model 3	1(ref)	1.15(0.74–1.79)	0.97(0.61–1.54)	1.12(0.71–1.79)	0.842	1.02(0.87–1.20)	0.842
**Sensitivity analysis**							
Model 4	1(ref)	1.13(0.73–1.76)	0.97(0.61–1.53)	1.08(0.68–1.73)	0.931	0.99(0.84–1.17)	0.931
Model 5	1(ref)	1.15(0.74–1.79)	0.94(0.59–1.49)	1.03(0.65–1.67)	0.887	0.99(0.83–1.17)	0.887

Model 1 unadjusted.

Model 2 adjusted for age (total + gender).

Model 3 adjusted for model 2 and smoking, BMI, SBP, FBG, TC, eGFR, MI, stroke, β-R inhibitor.

Model 4 adjusted for model 3 and further excluded MI.

Model 5 adjusted for model 3 and further excluded MI and stroke.

BMI, body mass index; MI, myocardial infarction; SBP, systolic blood pressure; FBG, fasting blood glucose; TC, total cholesterol; eGFR, estimated glomerular filtration rate; PVC, premature ventricular contraction; Hs-CRP, high-sensitivity c reactive protein.

## Discussion

In this prospective cohort study based on community populations, we found that elevated baseline hs-CRP was an independent risk factor for PVC risk. The association between increased baseline hs-CRP and subsequent PVC events remained sigfnicaint in men and in those without history of myocardial infarction or stroke. Result of our study may be reflective of the pathophysiological association between systematic inflammation and risk of PVC.

In our study, we were not able to detect a significant correlation between hs-CRP and PVC in women. One possible reason was the relatively low proportion of women in this cohort study which may limit the statistical power. One reason might be the ethnic heterogeneity which may suggest the different association between inflammation and PVC in different populations^19^. Another important reason likely was the difference of age-related risk patterns in men and women. A recent review study showed that women seemed to have a lower risk of cardiovascular disease until older age as compared to men. The potential mechanism might be related to the iron loss/deficiency during fertile years due to menstruation^20^. In the present study, we enrolled a total of 14010 women with a mean age of 48.15 ± 10.99 years old, which were relatively young. Hence, the relative protection from cardiovascular disease in fertile women probably contributed to the non-significant relationship between hs-CRP and PVC. In the sensitivity analysis, the relationship remained significance in participants without myocardial infarction or stroke. Of interest, we found lower hs-CRP concentration was a predictor of PVC incidence in men without myocardial infarction or stroke, compared with hs-CRP concentration in predicting further PVC in the whole population. It implied a cut off of hs-CRP concentration specificity in the particular population.

Results of our study may be reflective of the role of systematic inflammation in the pathogenesis of PVCs. It has been proposed that hs-CRP as a biomarker of inflammation, could stimulate the recruitment of monocytes into atheromatous plaque and induce endothelial dysfunction by suppressing basal and induced nitric oxide release, and finally contributes to the atherosclerotic process^21,22^. Recently, hs-CRP has been suggested to be of prognostic predictor in participants with CAD as well as in general populations^23–25^. Moreover, higher plasma hs-CRP has also been proposed as an independent risk factor of myocardial infarction, stroke, peripheral arterial disease and atrial fibrillation recurrence^26–28^. Our results suggest that hs-CRP was a significant predictor of PVC incidence, which were consistent with the previous findings of Biasucci *et al*.^29^, who found that hs-CRP concentration >3 mg/l is significantly associated with the occurrence of ventricular tachycardia (VT) or ventricular fibrillation (VF) in patients with implantable cardioverter defibrillator (ICD). Moreover, Blangy *et al*.^30^ also showed that increased hs-CRP was associated with a higher VT incidence. Indeed, Bonny *et al*.^31^ found that systemic inflammation expressed by high CRP concentration was a more active process after spontaneous ventricular arrhythmia in arrhythmogenic right ventricular cardiomyopathy (ARVC) patients. Furthermore, hs-CRP has also been suggested as a useful indicator of long term risk of sudden cardiac death in healthy men^32^, and in patients with chronic heart failure and reduced LVEF^33^. Interestingly, sudden death is reported to be mainly related to acute coronary event, but only isolated areas of myocardial fibrosis could be found in an autopsy case, suggesting that these areas might provide a myocardial substrate prone to develop arrhythmias^22^. Additionally, recent studies showed that elevated hs-CRP participated in atrial fibrillation recurrence via leading to electrophysiological and structural remodeling in the atrial and ventricular myocardium^10,34,35^. Hence, it could be speculated that elevated hs-CRP may accelerate the pathogenesis of ventricular arrhythmia via inducing of myocardial fibrosis. These facts may be reflective the potential mechanisms of statins administration for the prevention of ventricular arrhythmia in patients with ICDs, which may involve the anti-fibrotic effects following the anti-inflammatory benefits of the medications^36^. Further researches should be conducted to confirm the detail mechanisms for PVC induced by elevated hs-CRP.

Several researches demonstrated a poor prognosis in population with frequent PVCs^9,37^, and increased burden of PVCs has been linked to left ventricular dysfunction^38–40^. Sajadieh *et al*.^41^ demonstrated a hs-CRP concentration > or = 2.5 mg/l was associated with a significantly higher risk of death and acute myocardial infarction. In the present study, higher hs-CRP was related to higher risk of PVC incidence. Further studies should clarify whether there were particular groups of subjects who would have a worse prognosis. For this purpose, long-term follow-up may be the first step forward.

### Limitations and strengths

Possible study limitations should be considered. Firstly, participants enrolled in our study were from the Kailuan Mine Corporation, which included more men than women. The representativeness of the study was limited by the higher proportion of male subjects. Secondly, the prognosis of subjects with PVC and higher hs-CRP concentration was not known, further follow-up are need to confirm the results. Notwithstanding these limitations, our survey has important strengths including its large sample size, population-based research and relatively long time of follow-up.

## Conclusion

Elevated hs-CRP was an independent predictor of PVC in a Chinese population, especially in men, which may be reflective of the pathophysiological association between systematic inflammation and risk of PVC.
